# Spatial geometry of stem cell proliferation in the adult hippocampus

**DOI:** 10.1038/s41598-018-21078-6

**Published:** 2018-02-21

**Authors:** Olga A. Mineyeva, Grigori Enikolopov, Alexei A. Koulakov

**Affiliations:** 1 Center for Developmental Genetics, Stony Brook University, Stony Brook, NY 11794 USA; 2 Department of Anesthesiology, Stony Brook University, Stony Brook, NY 11794 USA; 3 Moscow Institute of Physics and Technology, Moscow, 123182 Russian Federation; 4 P.K. Anokhin Institute of Normal Physiology, Moscow, 125315 Russian Federation; 5 Kurchatov Institute National Research Center, Moscow, 123182 Russian Federation; 6 Cold Spring Harbor Laboratory, Cold Spring Harbor, NY 11724 USA

## Abstract

The modes of stem cell divisions (e.g., symmetric vs. asymmetric) can have a profound impact on the number of progeny and tissue growth, repair, and function. This is particularly relevant for adult neural stem cells, since stem cell-derived neurons affect cognitive and mental states, resistance to stress and disease, and response to therapies. Here we show that although dividing stem cells in the adult hippocampus display a certain bias towards paired distribution (which could imply the prevalence of symmetric divisions), this bias already exists in the distribution of the general population of stem cells and may be responsible for the perceived occurrence of symmetric stem cell divisions. Remarkably, the bias in the distribution of stem cells decreases with age. Our results argue that the preexisting bias in stem cell distribution may affect current assumptions regarding stem cell division and fate as well as conjectures on the prospects of brain repair and rejuvenation.

## Introduction

New neurons are continuously generated in selected regions of the adult brain. Production of new adult neurons starts with the activation and division of resident neural stem cells^1–3^. In the hippocampus, these stem cells are located in a narrow region (subgranular zone, SGZ) of the dentate gyrus (DG). Adult stem cells are marked by a long radial process that traverses the granule cell layer (GCL) and terminates with an arbor of fine processes in the molecular layer (ML). These cells can be identified directly, through examination of the expression of specific markers, application of viral labeling, or the use of transgenic reporter lines; they can also be identified indirectly, e.g., through lineage tracing or clonal analysis. These approaches are often combined with the labeling of nascent DNA with thymidine analogs. Hippocampal stem cells are mainly quiescent but can be activated to produce neuronal and astrocytic progeny^4–11^. Potentially, stem cells can undergo symmetric divisions (producing two copies of themselves), asymmetric divisions (producing one copy of themselves and morphologically or functionally distinct progeny), or engage a combination of these two modes. Using lineage tracing supported by proliferation analysis, we have previously found that, under normal conditions, the stem cells of the DG predominantly undergo asymmetric divisions and that activation of quiescent stem cells results in their subsequent conversion into regular astrocytes and disappearance from the stem cell pool^11^.

Our model sets forth asymmetric divisions as the prevalent mode of stem cell division in the adult hippocampus. This model also implies the gradual depletion of the stem cell pool. Moreover, it predicts that excessive activation of stem cells may lead to an accelerated decrease of the pool. By contrast, symmetric divisions may prevent the decrease of the stem cell pool and even lead to an increase. Given the importance of adult hippocampal neurogenesis for cognitive function^1–3,12–15^, determining the prevalent mode of neural stem cell division is essential for understanding both the biology of stem cells and their therapeutic potential^16^.

One possible approach to detect symmetric divisions of stem cells is to label dividing cells with a nucleotide analog and search for pairs of closely positioned labeled cells. In an orthogonal approach, one can genetically label dividing cells and determine the occurrence of pairs of stem cells within the same clone. To avoid false positives, both approaches require a correction that would estimate the probability of two dividing cells being located close to each other simply by chance. It is usually assumed in such analyses that individual neural stem cells, whether dividing or not, are distributed randomly, at least within small subdomains of the DG (larger subdivisions, e.g., dorsal vs. ventral hippocampus notwithstanding). Therefore, an observed bias towards unusually closely located cells, labeled biochemically or genetically, is interpreted as a strong indication of a recent symmetric division.

Although the assumption of randomness is crucial for understanding the basic mechanisms of the stem cell maintenance, it has never been rigorously tested; likewise, the potential biases in stem cell distribution and division have never been compared. Here we examine the spatial geometry of neural stem cell distribution and division in the adult DG and show that even when bias in the distribution of dividing stem cells is observed, it can be explained solely as the bias in the distribution of all (dividing and nondividing) stem cells. Moreover, we show that age-dependent disappearance of stem cells tends to randomize the distribution of the remaining cells in the DG. Our results call for a critical reevaluation of the current paradigms regarding symmetric vs. asymmetric divisions of stem cells (whether probed by DNA labeling or by clonal analysis) and, by extension, of stem cell loss due to aging and disease, as well as for a reassessment of the prospects of brain rejuvenation.

## Results

### Spatial distribution of dividing stem cells in the adult hippocampus

We first asked whether closely positioned dividing or recently divided neural stem cells may reflect a duplication of a common precursor cell. Detecting true pairs of related dividing cells can be challenging because the overall population of stem cells may display pre-existing pairing correlations in their positions. In other words, two recently duplicated stem cells can be found near each other for at least two reasons. First, it is possible that these two cells have indeed been produced from a common precursor that has recently undergone duplication. This scenario implies that adult stem cells can divide symmetrically. Alternatively, it is possible that those two cells have emerged from two separate precursors that were located near each other and therefore appear closer more frequently than a random distribution pattern would yield. In the latter scenario, pairs of closely positioned recently divided stem cells would reflect pairings pertinent to the initial location of those stem cells (which can be due to a number of reasons, including, but not limited to, nonrandom positioning during embryonic development, selective elimination during development and adulthood, or a common nearby blood vessel). Therefore, a relevant question is not whether close positioning of two dividing stem cells is different from random positioning, but whether it is different from the distribution pattern of the entire population of stem cells; i.e., it is critical to determine whether any non-random spatial bias in the distribution of dividing cells reflects a pre-existing bias in the distribution of their precursors.

Determining the deviation from randomness for the entire population of stem cells or its dividing subpopulation is challenging because of the difficulties in formulating the null hypothesis. The main reason is that a set of random stem cell positions has to be generated in three dimensions along the DG, which is problematic because the stem cell distribution along the DG is likely to be uneven. An additional difficulty is that even if the respective biases (deviation from random) of the entire pool and of the dividing subpopulations exist, it is not clear how these respective biases are related to each other. Therefore, rather than trying to directly determine the degree of randomness of the distribution of all stem cells and of dividing stem cells, we applied an approach based on a generative computational model, comparing the distributions of dividing stem cells and of all stem cells in the DG and evaluating their similarity.

We first determined the number and the 3D-positions of all (both quiescent and dividing) stem cells in the SGZ of young adult (2.5 months) mice, relying on the expression of the reporter transgene Nestin-GFP and of the stem cell marker GFAP to define radial glia-like (RGL) stem cells (this class largely overlaps with adult neural stem cells defined as Type-1^8^ or quiescent neural progenitors, QNP^6^). We next determined the positions of dividing stem cells after marking their progression through the S phase with a thymidine analogue 5-bromo-2′-deoxyuridine (BrdU). To ensure that the majority of cells traversing the S phase are labeled and to increase the number of cells scored as BrdU-positive, we infused BrdU for 5 days using osmotic minipumps. This protocol produced 777 ± 146 (value ± SD here and later) BrdU-labeled RGL cells per DG (cf. ~100 after one injection) within the pool of 8197 ± 195 all RGLs.

Importantly, the number of labeled RGL cells did not increase 1, 2, or 5 days after labeling (data not shown), indicating that the bulk of the stem cell population did not undergo symmetric division (which would have increased the number of labeled stem cells). However, even if the predominant fraction of stem cells did not engage into the symmetric division mode, it is possible that a smaller fraction did (i.e., a moderate increase, rather than doubling, in the number of labeled stem cells might not have been scored as significant). Therefore, we sought to examine the distances between BrdU-labeled stem cells in the DG and to determine whether there are pairs of closely positioned cells present. As noted above, if two labeled stem cells are located in closer proximity than expected for a random distribution, it would be anticipated that these cells have emerged as a result of a symmetric division or that two unrelated closely positioned precursor cells have entered the division cycle. Another possibility is that a stem cell may have divided asymmetrically, producing one labeled stem cell and one or more progeny; however this progeny does not possess the morphological or immunocytochemical characteristics of an RGL cell and, therefore, will not be scored as a stem cell.

To help disambiguate between these scenarios, we formulated a quantitative model for stem cell activation and division that we call the random activation model (RAM). In RAM, RGL stem cells mostly reside in a quiescent (G0) state. Occasionally, a stem cell enters the cell cycle (G1 phase) and divides asymmetrically, producing an RGL stem cell and its morphologically differing progeny (transit amplifying progenitors). Within RAM, activation of a stem cell is fully random, i.e. a cell can enter the division cycle at a random moment in time and independently of other cells. Also, to increase the stringency of our modeling, we assume that RAM includes asymmetric, but not symmetric divisions. We next asked whether the observed distribution of dividing (BrdU-positive) stem cells in the DG is consistent with RAM.

**Figure 1:**
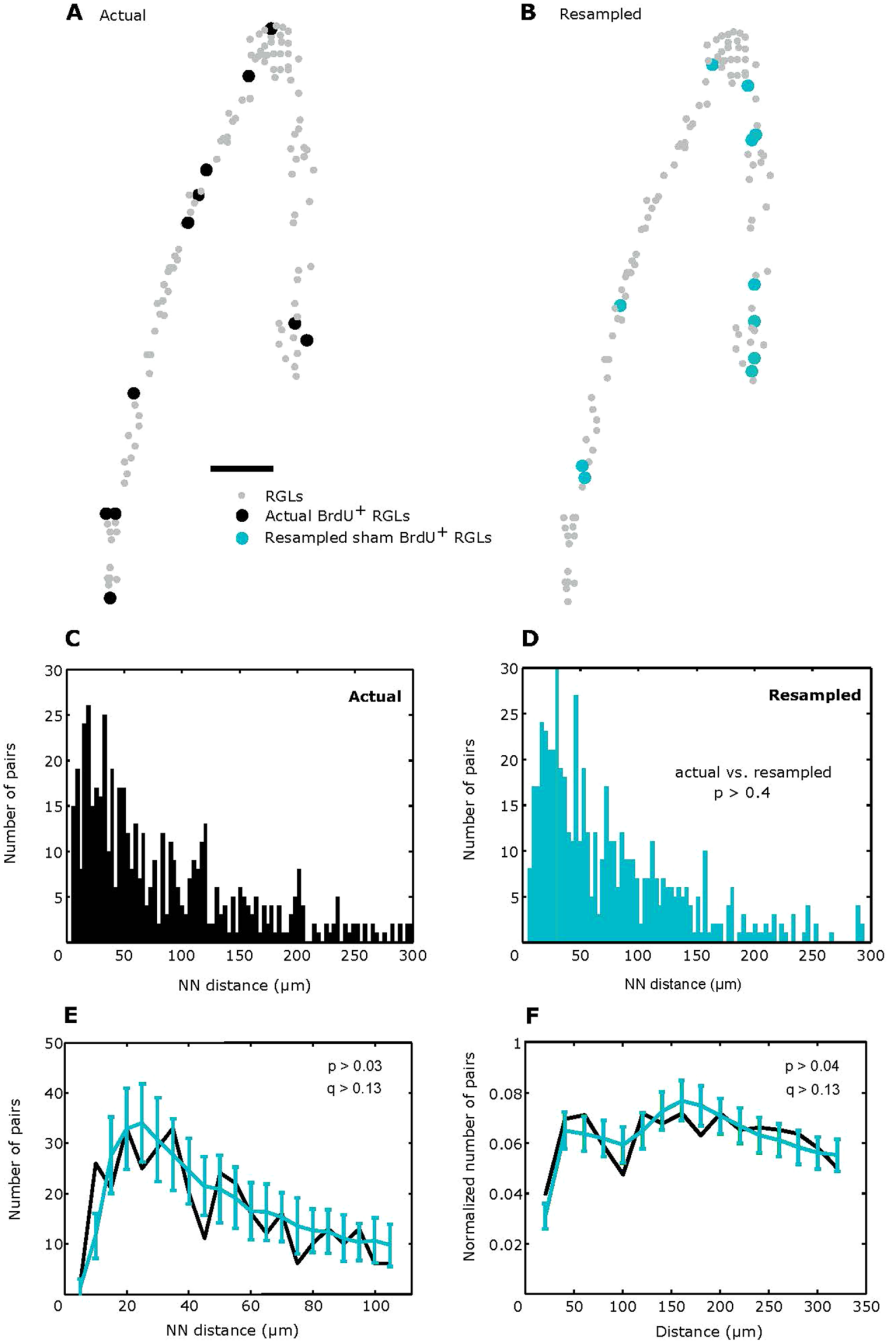
The distribution of distances between the nearest dividing cells is not different from random. (**A**) Actual positions of all RGLs (grey dots) and BrdU^+^ RGLs (black dots) in a single section from 2.5 month old mouse. This section contained 11 BrdU^+^ RGLs. (**B**) Positions of resampled sham BrdU^+^ RGLs (blue dots) within the same actual positions of all RGLs. For the resampling procedure in this section, we randomly selected N = 11 RGL cells designated as sham labeled cells. Scale bar: 100 μm. (**C**,**D**) The distributions of nearest neighbor (NN) distances between the centers of actual BrdU^+^ and resampled sham BrdU^+^ RGLs. *p* > *0.4* calculated with the Kolmogorov-Smirnov test (see Methods). Calculated distances from 74 sections of *n* = *4* animals were pooled for direct measurement of the distribution of BrdU^+^ cells (**c**), and the calculated distances from equal numbers of randomly selected sections from the same animals were pooled to obtain the distribution of resampled or sham BrdU^+^ RGLs (**D**) (see Methods). (**E**) The distribution of NN distances with 5 μm bins. The black line shows the actual distribution (same data as (**C**), <100 um range) and blue line represents the resampled distribution obtained from 1,000 random samplings. The center values and error bars represent the means and SD. *p* > *0.03* and *q* > *0.13* for every bin, computed using bootstrap and FDR correction for multiple hypothesis (see Methods). (**F**) The same analysis for all distances (not necessarily nearest) between actual (black) and randomly resampled (blue) RGL cells. Bin size is 20 μm. *p* > *0.04*, *q* > *0.12* for every bin.

We used the information that we obtained on the 3D distribution of the entire pool of RGL stem cells to generate a simulated distribution of their dividing subset consistent with RAM. To that end, we applied a resampling method, using the coordinates of the entire RGL population and random number generator to assign BrdU^+^ status to some of the DG stem cells. Thus, to generate the positions of sham BrdU^+^ stem cells conforming to the RAM model, we selected a random subset of the general population of RGL stem cells (Fig. 1A,B). For each slice, the number of randomly chosen cells was selected to match the number of BrdU^+^ stem cells in that slice and the procedure was repeated multiple times in a bootstrap algorithm. The distributions of the nearest neighbor (NN) distances for the true BrdU^+^ stem cells and for the “sham” dividing stem cells, randomly selected (resampled) according to RAM, are shown in Fig. 1C and D, respectively. The NN distance distributions were not statistically different, according to the Kolmogorov-Smirnov test, between the observed (Fig. 1C) data and the resampled (sham) set (Fig. 1D). Thus, dividing (BrdU^+^) stem cells are not found in pairs more frequently than what would be expected in the random model of stem cell activation.

To further test the hypothesis of a non-random distribution of dividing stem cells in DG, we analyzed the number of RGL cells found at specific distances from each other (Fig. 1E,F). To this end we compared both the NN distance distribution and the distribution of all RGL cell pairs (i.e., not necessarily the nearest) bin-by-bin, with a bin size of 5 μm and 20 μm, respectively. To detect significant differences in such multiple comparisons, care should be taken regarding testing multiple hypotheses. Indeed, even for fully random data, when multiple hypotheses are tested, some are expected to yield p-values that fall below the significance level. Because we perform comparisons bin-by-bin, it is possible that comparison of individual distance ranges could yield small p-values, even if the distributions are random (Fig. 1E,F). A false discovery rate correction for multiple hypothesis testing yields q-values that were well over significance levels (q > 0.13) for both the NN distances and all cell-to-cell distances (Fig. 1E,F). Thus, as with the previous comparison (Fig. 1A,B), the number of dividing cells found within individual distance ranges is not different from the random model. Overall, our data indicates that the distribution of dividing RGL stem cells in the adult hippocampus is not different from that expected from the random model (RAM) that does not invoke symmetric cell divisions.

While our results are consistent with the fully asymmetric mode of RGL divisions, we asked how sensitive is our method to the occurrence of symmetric divisions in our system. To address this, we generated, based on the data on positions of BrdU-positive and all RGLs, simulated datasets which included variable fractions of symmetrically-dividing RGL cells. We selected a certain number of close RBL pairs to represent simulated daughters of the cells that have recently divided symmetrically. The distance between such cells is expected to be smaller than the distance between arbitrarily selected asymmetrically-dividing RGLs in the same volume. The simulated pairs of daughter RGLs were therefore selected from the lists of closest pairs of RGLs in each volume (regardless of their BrdU status) and assigned as sham BrdU-labeled cells. The rest of sham BrdU-positive cells were selected from the remainder of RGLs in that volume and are expected to represent asymmetrically-dividing RGLs (this was validated by our observation that the experimentally observed spatial distribution of BrdU-positive cells is not different from random, Fig. 1). We then applied our resampling procedure to the artificially generated datasets containing a controlled number of simulated symmetric divisions (Fig. S1) finding that if the fraction of symmetric divisions is close to 5%, out of 1000 artificial cell distributions, ~30% would yield p values larger than observed by us (p = 0.4, Kolmogorov-Smirnov test, Fig. 1C,D), if the fraction of simulated symmetric divisions was 10% of all divisions, we would observe p > 0.4 in 7% of cases, and with 15% fraction – only in 1% of cases Fig. S1). Thus, our results indicate that if symmetric divisions constitute above 10% of the division events, they are expected to be reliably detected by our assumption-free random resampling technique. In summary, our results indicate that the apparent spatial pairings of dividing RGL cells can be explained by the pre-existing pairings of non-dividing RGL cells in the DG, without invoking symmetric divisions.

We next sought to account for cells in the DG that may have stem cell potential but do not display the overt RGL phenotype. We first analyzed GFP-expressing cells that resemble RGL cells but whose GFAP-positive process was either horizontal, or angled, or directed towards the hilus (excluding cells in the lateral-most section where the DG blades do not form a regular V-shape; RGL cells at the point of blades connection where the direction of the apical process may be difficult to discern; and cells in the regions where the SGZ plane is angled to the sagittal cutting plane)^17,18^. The number of such cells was over 50-fold lower than that of RGL cells (Fig. S2). Within this group, the fraction of dividing cells was higher than for RGL cells (0.38 vs. 0.08). Thus, these RGL-resembling cells would constitute a small fraction of all RGL cells (~2%) or of dividing RGL cells (~8%). To evaluate whether these cells undergo symmetric divisions, we searched for pairs of BrdU-labeled RGL-resembling cells formed with the similar cells or RGL cells. We found on average only 6 pairs formed by labeled RGL-resembling cells and 6 pairs of RGL-resembling/RGL labeled cells, located within 50 µm, per animal. We also searched for BrdU-labeled GFAP-expressing cells in the SGZ that do not express GFP, finding only 15 such cells, forming 3 pairs, per brain. Thus, other analyzed stem-like cell types do not make significant contribution to our analysis and conclusions.

### Age-dependent decline in the RGL population

**Figure 2:**
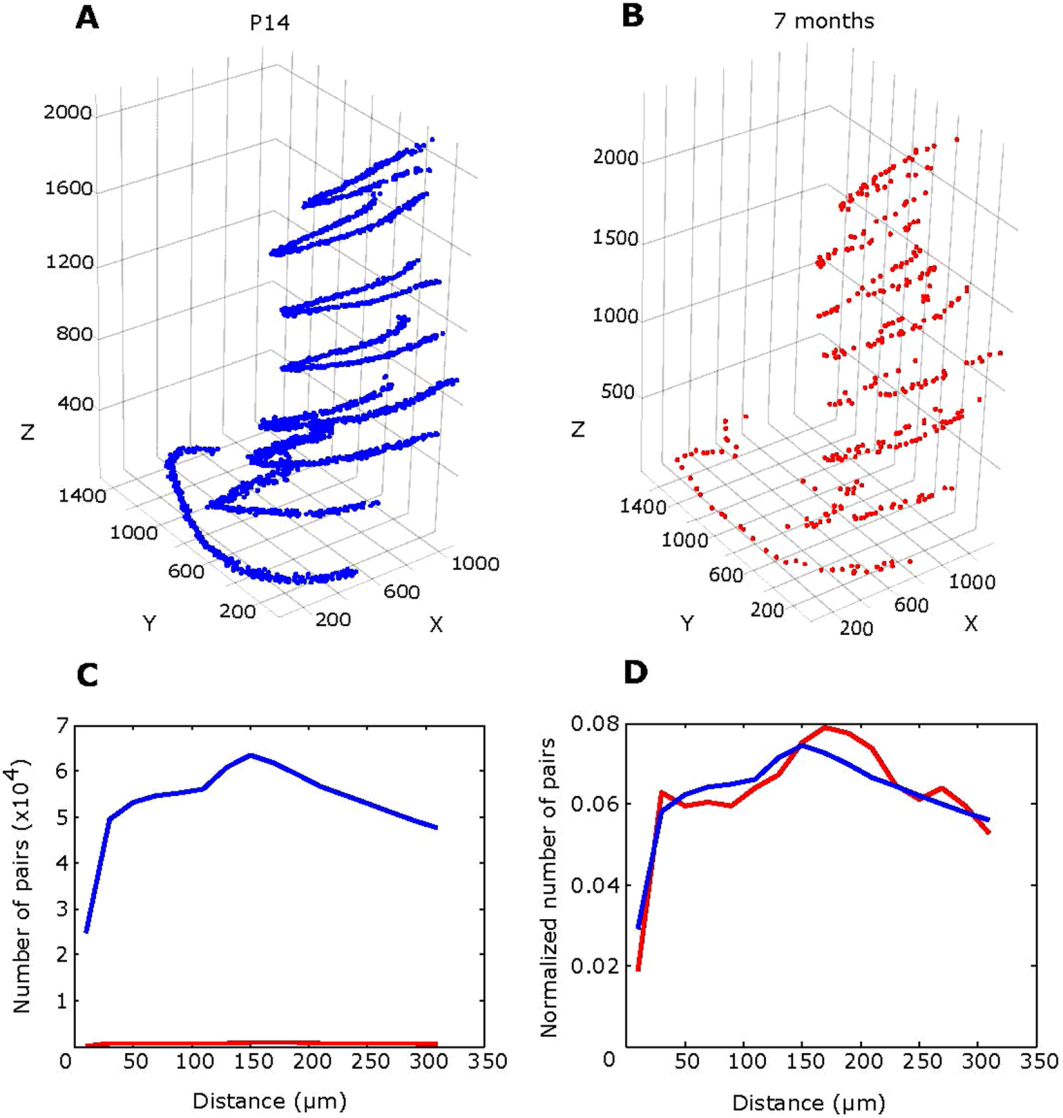
Age-dependent depletion of stem cells within 6.5 months. Stem cells’ distribution in serial sections of young and adult mice. Sagittal brain sections were collected every 300 μm, resulting in *n* = 6 slices (50 μm width) for a P14 mouse (**A**) and *n* = 8 sections for a 7 month old mouse (**B**). The slices are stacked sequentially as collected, from lateral (lower sections in the stack) to medial (top sections) side of one hemisphere. Substantially more stem cells were found in younger mice. In addition, the overall size of DG was found to be ~15% larger in older mice. Absolute (**C**) and normalized (**D**) numbers of cell pairs in different ranges of distances, in P14 (*n* = 4, 26 sections, blue line) and 7 month old mice (*n* = 4, 31 sections, red line).

Our previous results indicated that the number of hippocampal RGL stem cells gradually declines with age due to their division-coupled astrocytic differentiation (Encinas *et al*.^11^). To compare the spatial distribution of RGL stem cells in the hippocampi of mice of different ages we analyzed the DGs of young (2 weeks old) and adult (7 month old) Nestin-GFP reporter mice, visualizing all RGL cells and identifying their position as described above. The hippocampi of older mice contained substantially fewer RGLs than those of younger animals, with a 9.1 fold decrease over 6.5 months (326 RGLs per section at P14 vs. 36 cells per section at 7 months) (Fig. 2).

An important factor to consider is that the size of the hippocampus undergoes a substantial increase between 2 weeks (P14) and 7 months of age. Therefore, we examined the magnitude of changes in the number of RGLs that can be potentially due to the size change. We found that in P14 animals a root mean square (rms) distance within pairs of RGLs is 397 µm, whereas in 7 month old animals the pairwise rms distance is 458 µm, a 1.15-fold increase. Because pairwise rms distances were calculated for all pairs of cells, not necessarily the nearest neighbors, this measurement is dominated by long distances and, as such, is a descriptor of the overall size of the DG. This argument is confirmed by the observed values of rms distances (397 and 458 µm for P14 and 7 month old animals, respectively). This observation suggests that the linear size of DG expands by 15% between the analyzed time points. Taking into account that the slice thickness was the same for the specimens of both ages and assuming that the age-related expansion of the hippocampus occurs uniformly in all three dimensions, the factor describing age-dependent decrease in RGL counts (here and in subsequent calculations) must be corrected by a factor of 1.15 to yield 9.1/1.15 = 7.8. Thus, our results show that the expansion in hippocampal size alone is insufficient to explain the depletion of RGL.

Given the striking age-related diminishment of the RGL cell pool, we assessed whether the disappearance of RGLs follows a particular pattern. As in the case of the dividing RGLs described above (Fig. 1), rather than determining the potential randomness of the spatial RGL distribution at different ages, we directly compared the patterns of RGL distributions at the ages in question.

**Figure 3:**
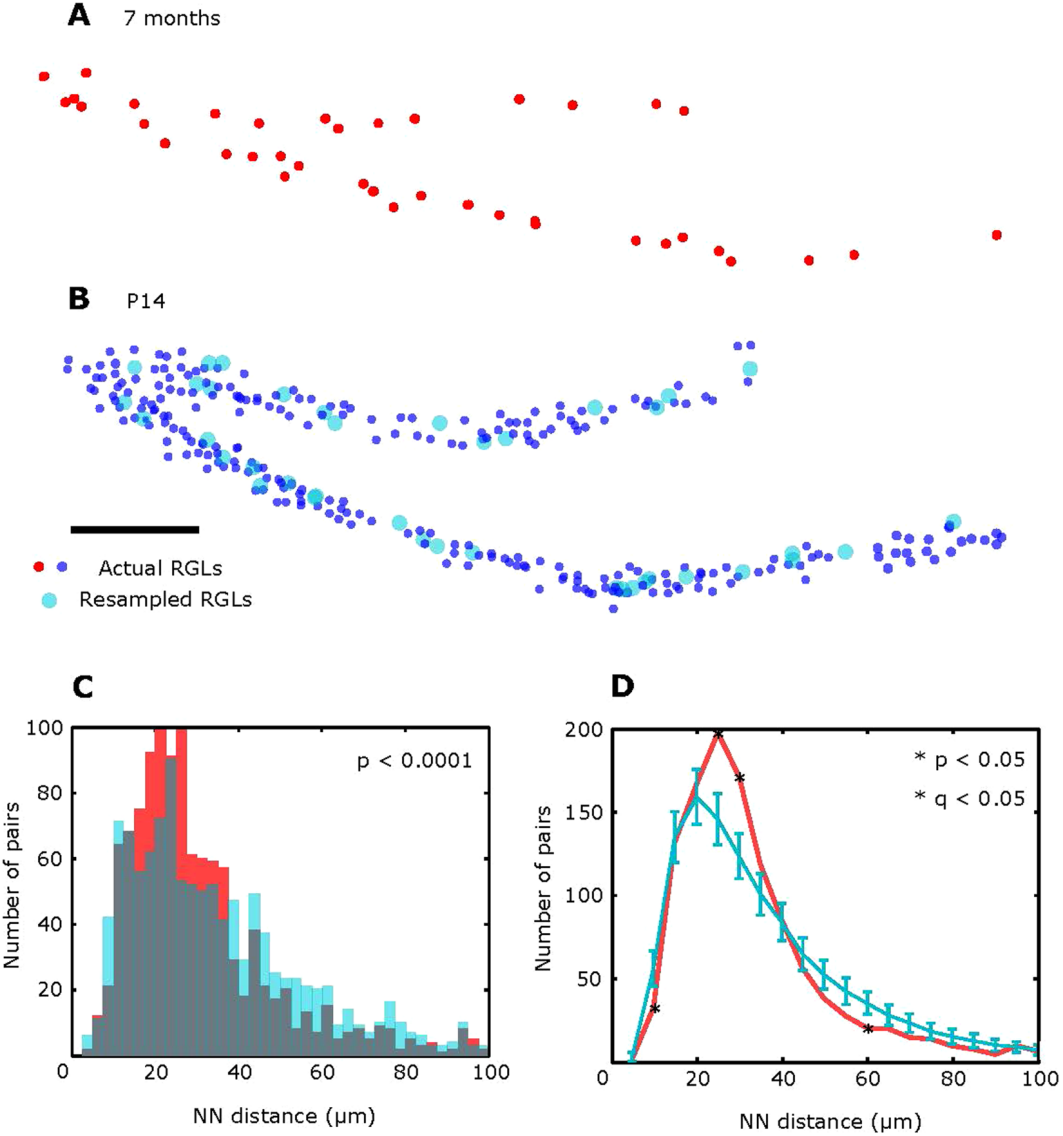
Age-dependent elimination of stem cells is non-random. All RGLs in a slice from a 7 month old animal (**A**) and of a P14 animal (**B**) (dark blue dots). Scale bar: 100 μm. The slice from a young mouse (**A**) was rescaled to compensate for the 15% expansion in the overall size of DG with age. Light blue dots in (**B**) represent a “sham” old section: a randomly sampled subset of all cells that is equal in number to the older DG presented in (**A**). (**C**) NN distributions for the actual RGLs (red, 31 sections from *n* = 4 7 month old mice) and resampled RGLs (blue, a random resampling of cells in 31 slices randomly chosen from 26 sections collected from *n* = 4 P14 mice), overlay represented in grey. P values were calculated with the Kolmogorov-Smirnov test. (**D**) The distribution of NN distances obtained from 1,000 random resamples (blue, mean and SD) and the distribution of actual NN distances (red) are shown. The results of bin-by-bin statistical analysis with adjustments for multiple comparisons (FDR) (see Methods) produced *p* = *0.003* and *q* = *0.02* for the 5–10 μm bin, *p* = *0* and *q* = *0* for the 20–35 μm bin, *p* = *0.001* and *q* = *0.01* for the 25–30 μm bin, and *p* = *0.006* and *q* = *0.032* for the 55–60 μm bin. Fewer cells are found at smaller distances in the real sections than in the generated sections after random elimination, suggesting that the process of stem cell elimination is biased towards small distances.

We asked whether age-related elimination of 87% of RGL cells is a random process that does not depend on cell positions. Taking the above correction factor into account, we compared the distribution of RGL cells in animals of different ages. To model the random process of elimination, we first chose a slice of hippocampus of an old animal (Fig. 3A) and a random slice from a young animal (Fig. 3B). We then eliminated a random subset of RGLs from the young animal’s brain section so that the final number of cells exactly matched the cell population in the adult animal’s section (see Methods for more detail). As in the experiments with dividing RGLs above, we performed the procedure repeatedly by bootstrapping. The resulting distribution of cells is expected to statistically represent the DG of the young animal at the older age (“sham” adult animals), generated through a random process of RGL elimination. We then compared the NN distribution in the “real” adult animals with that in “sham” adult animals (i.e., whose patterns were derived from the young patterns by random elimination).

Our analysis shows that the distributions of NN distances are different in the DG of adult animals and of “sham” adult animals, whose pattern was produced by random elimination (Fig. 3C, Kolmogorov-Smirnov p < 10^−4^). As with the previous series of experiments, we performed an increasingly detailed analysis of bin-by-bin differences between the two distributions using a combination of bootstrapping to evaluate p-values and False Discovery Rate (FDR) to account for multiple hypothesis testing by computing q-values. Our analysis shows that the number of cell pairs at short distances (5–10 µm) is smaller in the actual (“real”) data compared to the randomly generated data for the sham group. Conversely, data in 7 month “real” adult animals show more cells at larger distances (20–30 µm). Consequently, the average NN distance is slightly larger for the real data than for the sham data (dots in Fig. 3D).

The depletion of cell pairs at small distances (5–10 µm) implies that the process of age-dependent cell elimination is different from random. Cells are more likely to be eliminated if the distances to their neighbors are smaller. Note that this observation is contrasting with the hypothesis of symmetric stem cell divisions, which should produce more pairs of cells at short distances than what would be expected for random. Overall, our data suggests that the process of age-dependent RGL elimination occurs through removal of cells with shorter distances to other cells at a higher rate, thus ensuring a more uniform spacing between cells that what would be expected at random.

### Stem cell disposal along the supra- and infrapyramidal blades and the septotemporal axis

**Figure 4:**
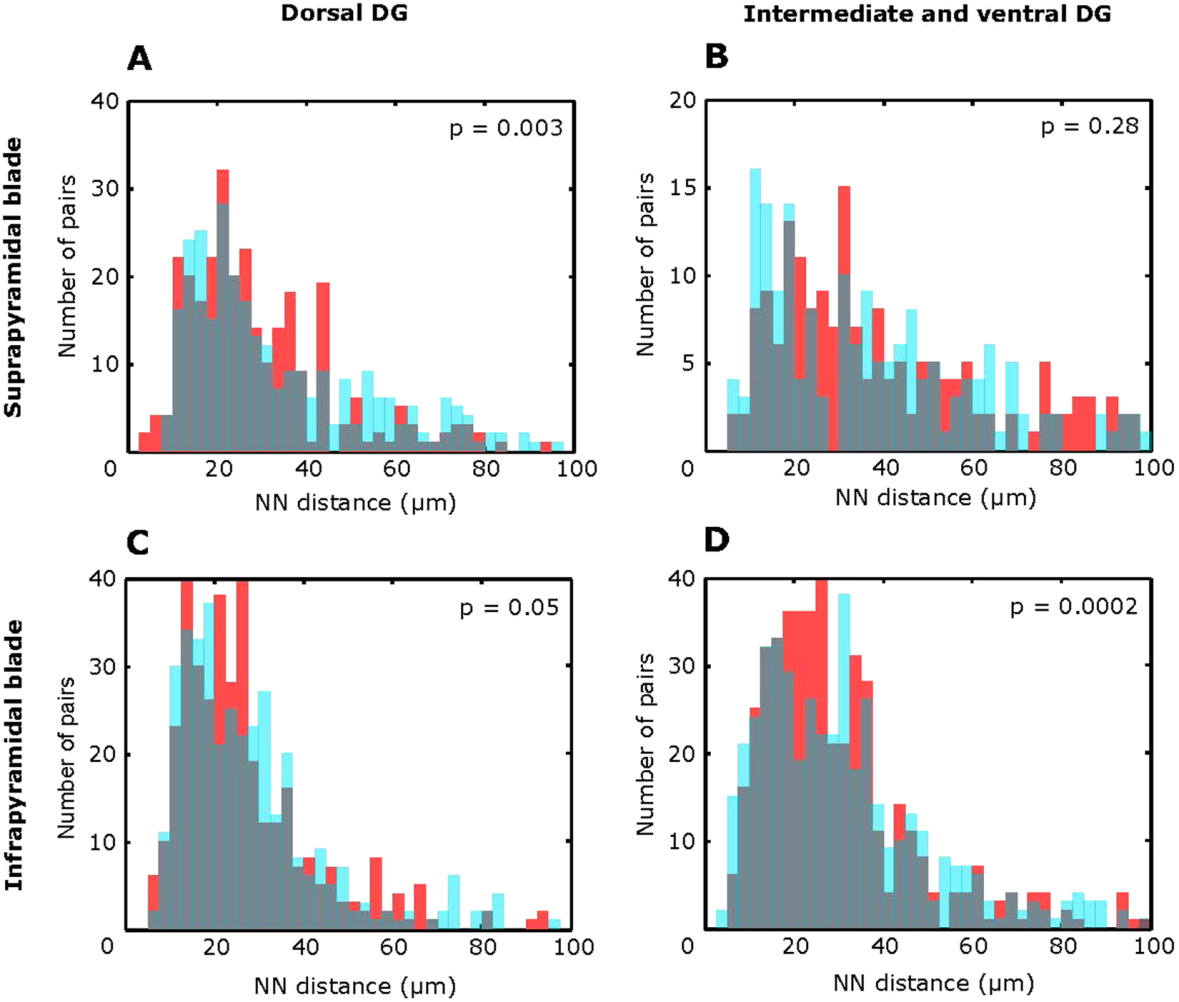
The mechanism of age-dependent stem cell elimination is not uniform. Cell elimination is random in the suprafrapyramidal blade of the intermediate/ventral DG (**B**), but is non-random across the infrapyramidal blade (**C**,**D**) and in the upper blade of the dorsal DG (**A**). 11 dorsal and 19 intermediate/ventral slices were obtained from P14 animals (*n* = 4) to visualize the actual distributions (red). 16 dorsal and 19 intermediate and ventral slices from 7 month old mice (n = 4) were used to randomly choose 11 dorsal and 18 intermediate/ventral sections and obtain 1,000 distributions for the resampled RGLs (examples in blue). An overlay of distributions is represented in grey. Each generated resampled distribution was compared to the actual results from within a region. For each comparison, p values were calculated with the Kolmogorov-Smirnov test, and their logarithmic values were then averaged (the resulting p values are shown).

To account for possible local non-randomness in stem cell elimination, we next asked whether age-dependent stem cell disposal differs between the infrapyramidal and suprapyramidal blades of the DG and along the septotemporal (dorso-ventral) axis^19^. The same process of random cell selection was applied to the DG of young mice within the defined regions and the distributions of NN distances of “real” RGLs in the adult brain (7 months) and of resampled “sham” RGLs in the young brain (2 weeks) were compared. This analysis revealed that the actual stem cell pool was eliminated in a non-random pattern in the infrapyramidal blade and in the dorsal part of the suprapyramidal blade (Fig. 4A,C,D, Kolmogorov-Smirnov test). However, stem cell loss was not different from random in the suprapyramidal blade within the combined intermediate and ventral DG (Fig. 4B, Kolmogorov-Smirnov test). These results indicate that age-dependent stem cell elimination can be influenced by regional factors when dissected by both major functionally distinct hippocampal subdivisions and smaller circuits.

## Discussion

Adult hippocampal neurogenesis is important for learning and memory, response to stress, and the action of antidepressants, among other functions. Production of new neurons decreases with age and disease and this decrease may underlie age- or disease-related decline in cognitive abilities. Increased production of new neurons may help to overcome or slow the effects of age and disease. Hence, significant effort has been directed toward the goal of augmented hippocampal neurogenesis, and various proposed strategies of brain rejuvenation rely on increased production, or decreased death, of hippocampal neurons. Therefore, the modes of maintenance and division of neuronal progenitors and the prospects of manipulating them are often the focus of strategies for improving the brain function.

We have previously proposed a model of stem cell maintenance and division where stem cells mainly undergo asymmetric divisions and, upon a series of divisions, convert to astrocytes, thus leaving the stem cell pool^11^. This model leads to several important predictions. One is that the profound age-related decrease in hippocampal neurogenesis may be due primarily to the gradual diminishment of the stem cell pool (rather than, for instance, a decrease in the capacity of a stem cell to produce neuronal progeny). Another prediction is that excessive activation of stem cells may lead to a premature exhaustion of their pool. Indeed, recent findings show that enhanced activation of adult neural stem cells by kainic acid-induced seizures results in an increased conversion of stem cells into astrocytes and their early depletion^20^.

The prospect for the exhaustion of the pool of neural stem cells due to their asymmetric divisions and subsequent differentiation highlights the importance of determining the true mode of stem cell maintenance and division^16,21^. Indeed, this issue has been addressed using various approaches^4,9–11,22,23^. However, these approaches, each with its own limitations, may lead to different conclusions regarding their division and fate. For instance, clonal analysis of stem cell division and differentiation is compatible with mainly symmetric divisions^4^, whereas pulse labeling with nucleotide analogs indicates that the predominant mode of stem cell division is asymmetric^11^. However, clonal analysis does not provide direct information on the division events, whereas pulse labeling may not account for cells that have a particularly protracted S phase or a long division cycle. Therefore, we sought to apply an alternative approach that would not focus on modes of division per se but would rather assess whether the results of clonal analysis or nucleotide labeling may be obscured by some pre-existing peculiarities of neural stem cell distribution. For instance, is it possible that there is a pre-existing bias for stem cells to be positioned in pairs or clusters of several cells?

Clearly, stem and progenitor cells may be found near each other for reasons other than their symmetric divisions immediately preceding observation. It is possible, for instance, that closely located cells originated from the same mother cells during neural development and remained adjacent to each other in the adult brain. It is also possible that cells were eliminated in the developing or adult brain in a non-random fashion (e.g., if cell elimination depends on their spatial arrangement). Another possibility is that the efficiency of recombination in lineage tracing experiments is not equal across the brain tissue and two neighboring cells are more likely to be marked by the tracer expression, especially when the rate of recombination is kept low to implement sparse labeling. The sheer adjacency of stem cells to a blood vessel may also introduce non-randomness and bias into their distribution, activation, labeling, or recombination.

Here we incorporate information regarding cells’ mitotic status into our analysis and examine whether pairs of dividing stem cells are found near each other more frequently than at random. To perform this analysis carefully, a model of a random distribution of dividing cells is required. This is problematic and may be misleading; for instance, if the cell placement shows a smooth septotemporal gradient (even without a bias in the distribution of cells in pairs or clusters), randomly distributing cells in a 3D model will produce an incorrect starting point. Moreover, the types of biases and unevenness may be different for the general and the dividing populations of stem cells. Our approach bypasses these potential complications and focuses instead on comparing the actual and the sampled (modeled) distributions of dividing stem cells across the hippocampus; this way the spatial biases present in the general population of stem cells are taken into account (since dividing cells are the subset of the latter).

Therefore, we performed the analysis of stem cell distribution in two steps. First, we generated an exhaustive 3D map of RGL stem cells in the adult hippocampus. Next, we generated a 3D map of the dividing subset of the RGL cells and compared it to an appropriately resampled subset of all RGLs.

Our results show that the distribution of the dividing cells is not distinguishable from the random activation model, i.e. a scenario in which stem cells enter mitosis without regard to their spatial location. Therefore, we conclude that there are no spatial biases in the distribution of dividing cells that cannot be explained by the spatial correlations in the general RGL population. In other words, dividing RGLs do not appear closer to each other than what is expected at random if the preexisting unevenness of cell distribution is taken into account; thus, a conclusion regarding pairs of RGLs appearing in close proximity to each other due to symmetric divisions is disproved by these results.

We applied a similar rationale to the issue of stem cell depletion. Since it is impossible to predict all of the existing biases in the stem cell distribution patterns at different ages, we generated a “randomly” aged brain by randomly eliminating cells from the young DG. Remarkably, we found that the distribution of stem cells in an aged brain is inconsistent with the model of random elimination. We find that stem cells disappear in such a fashion that cells located close to other stem cells are more likely to be eliminated, making stem cell spatial distribution more uniform with age. In particular, we find that the number of cells at short distances from each other is smaller than what would be expected from random elimination. We hypothesize that the process of cell elimination may be a niche-determined mirror image of cell birth and activation; for instance, if a pair of cells is positioned closely due to their common origin, activation and subsequent elimination of one of them may be more likely. Moreover, the depletion process is uneven across septotemporal and transverse axes and implies either inherent or developed heterogeneity of stem cell distribution.

Our findings highlight the distinction between two specific hypotheses, - on the disposable stem cell and on the asymmetric division cascade. The former hypothesis posits that quiescent stem cells, once activated, produce new neurons and are terminally removed from the pool of potential, quiescent, or active neuroprogenitors. The latter hypothesis suggests that quiescent stem cells, once mitotic, undergo asymmetric divisions for the most part. These two hypotheses do not mutually exclude each other. For example, a stem cell could undergo a series of symmetric divisions, after which all progeny of this cell with neurogenic capacity would leave the stem cell pool; similarly, a stem cell could continue to divide asymmetrically without leaving the pool. Thus, demonstrating symmetric divisions does not disprove the disposable stem cell hypothesis. Similarly, showing that quiescent stem cells predominantly undergo asymmetric divisions, does not prove that they are disposable. Our current results do not shed further light on the disposable stem cell model; however, they provide strong support for the asymmetric mode of stem cell divisions. We have shown that the spatial distribution of dividing stem cells is fully consistent with the model in which the bulk of cells divides only asymmetrically. Symmetric divisions, if present, should yield pairs of cells in the S phase within a short distance from each other. The presence of such pairs of mitotic cell population should be detectable, if this involves a significant fraction of the stem cell pool. Our data, however, shows no anomaly in the nearest neighbor distribution that is inconsistent with the model of asymmetric divisions. As such, our findings rule out a substantial presence of symmetric stem cell divisions and confirm the predominantly asymmetric mode of neural stem cell division.

Besides clarifying the mechanisms of neural stem cells maintenance and division and highlighting the limitations inherent to various methods of stem cell analysis, our conclusions are relevant in the broader issue of stem cell role in brain function and the prospects for neural tissue repair and rejuvenation. A possibility of switching to a particular and preferential mode of stem cell division depending on cognitive and emotional state, disease, and environment has been proposed^2,4,20,22,23^. Our findings emphasize the importance of a broader search for agents that can induce symmetric division of neural stem cells as means of repairing or rejuvenating the damaged or aging brain.

## Methods

### Animals and BrdU labeling

For long-term labeling of dividing cells in 2.5 months old male heterozygote Nestin-GFP mice^6^, BrdU (Sigma) was diluted in sterile saline to obtain 50 mg/ml solution^24^ and transferred to cylindrical osmotic infusion pumps (Model 1007D of Alzet Micro-Osmotic Pumps, Durect Corporation, Palo Alto, CA). As described in^24^, to minimize the delay between implantation and the onset of release, the filled pumps were incubated in PBS at 37 °C overnight before implantation. Pumps were implanted subcutaneously for 5 days (3 mg of BrdU diffusing into each animal). All mice were monitored daily for the general health during the infusion period. After 5 days, the pumps were removed and mice were sacrificed immediately (n = 4) for the evaluation of the stem cell positioning. 3 additional groups of animals were analyzed at 1, 2 and 5 days after the end of infusion (n = 4 for each group, except for 5 days with n = 3) to check if the number of labeled stem cells increased with time. To study the distribution of RGLs in young and middle-aged brains, heterozygote Nestin-GFP mice of P14 (n = 4) and 7-months (n = 4) were used with no thymidine analogue administration. Sample sizes were chosen in accordance with our previous experiments with counting the number of all RGLs and pulse-labeled RGLs^11^. Mice were randomly assigned to experimental groups and no animals (or their brain sections) were excluded from the analysis. 2.5 month old mice were housed in the animal facility of Cold Spring Harbor Laboratory (NY, USA), P14 and 7 months old mice were kept in the animal facility at NRC Kurchatov Institute (Moscow, Russia), in identical conditions (a standard light- and temperature-controlled environment, 12-hour light/dark cycle; light on at 7:00 AM; 21 °C, with access to food and water ad libitum). Mouse maintenance followed the guidelines for the use and treatment of laboratory animals from the National Institutes of Health and the European Communities Council Directive (86/609/EEC). All procedures were approved by Animal Care and Use Committees of Cold Spring Harbor Laboratory and Biochemical Research Committee of NRC Kurchatov Institute.

### Immunohistochemistry

Animals were subjected to transcardial perfusion with 30 ml of PBS followed by 30 ml of 4% (w/v) paraformaldehyde in PBS, pH 7.4. The brains were removed and postfixed with the same fixative overnight at 4 °C, then transferred to PBS and kept at 4 °C. Before sectioning the brains were cut longitudinally into two hemispheres. Serial 50 µm thick sagittal sections were cut using Vibratome 1500 (Vibratome) or VT1200S (Leica). For BrdU-labelling and RGLs’ distribution analysis in 2.5 month-old mice, immunostaining for BrdU, GFAP, and GFP was carried out in floating sections. For RGLs distribution analysis in P14 and 7 month-old mice, immunostaining was carried out for GFAP and GFP. The sections were incubated with blocking and permeabilization solution (PBS containing 2% Triton-100X and 5% normal goat serum (Abcam, ab7481) for 1hr at room temperature. Sections destined to the analysis of BrdU incorporation were treated, before the immunostaining procedure, with 2 M HCl for 30 min at 55 °C and rinsed with PBS in triplicate, at room temperature. Next, all sections were incubated at 4 °C overnight with primary antibodies diluted in 0.2% Triton-100X in PBS with 3% goat serum. After washings with PBS, the sections were incubated with fluorochrome-conjugated secondary antibodies diluted in 0.2% Triton-100X in PBS with 3% goat serum for 2 hrs at room temperature. After washings with PBS, the sections were mounted on uncoated slides with Fluorescent Mounting Medium (DAKO, S3023) and dried at room temperature overnight. The following antibodies were used: chicken anti-GFP (Aves Laboratories, GFP-1020) at 1:500 dilution; mouse anti-BrdU (MoBU-1 clone, Invitrogen, B35128) at 1:400; rabbit anti-GFAP (DAKO, Z0034) at 1:500; AlexaFluor 488 goat anti-chicken (Invitrogen, A11039) at 1:500; AlexaFluor goat anti-mouse 633 (Invitrogen, A21052) at 1:500; AlexaFluor 405 goat anti-rabbit (Invitrogen, A31556) at 1:500 or AlexaFluor 647 goat anti-rabbit (Invitrogen, A21245) at 1:1000.

### Imaging

Immunostained sections from 2.5 month old mice were imaged with a PerkinElmer UltraVIEW Vox epifluorescence/bright field spinning disk confocal microscope (PerkinElmer, 40X objective air lens) equipped with the Volocity 6.0.1 software (PerkinElmer). Sections of P14 and 7 month old mice were imaged with an Andor Revolution WD spinning disc microscope (Andor, Ireland, 40X objective air lens, iQ 3.1 software (Andor)). Optical serial Z stacks were obtained at 1μm intervals. The signal from each fluorochrome was collected sequentially. Images collected with iQ3.1 were stitched together with XuvStitch software^25^. All images were imported into Imaris 7.6.4 (Bitplane, Switzerland) in tiff or ims format.

### Quantification

Quantitative analysis of cell populations was performed by means of design-based (assumption free, unbiased) stereology using a modified optical fractionator sampling scheme as previously described^11,26^. Slices were collected using systematic-random sampling. One brain hemisphere was randomly selected per animal. The hemisphere was sliced sagittally in the lateral-to-medial direction, from the beginning of the lateral ventricle to the middle line, including the entire DG. All 50 µm slices were collected 300 µm apart. For each experiment (BrdU tracing and age-dependent stem cell elimination analysis), the investigators were blinded during data collection and cell quantification. BrdU-labelled cells were categorized as RGL cells if positive for Nestin-GFP and GFAP with a process extending from the SGZ until at least the third quarter of the granule cell layer, without branching in the two first quarters of the total length of the GCL. The same stereological procedure was followed for the experiments on age-dependent changes in the number of hippocampal RGLs (P14 and 7 months old Nestin-GFP mice, one set of slices analyzed). Quantification was restricted to the SGZ and was achieved by several image view options, including extended focus, Z planes, and 3D-rotation in Imaris (Bitplane). A center of each RGL cell (with or without BrdU labeling) was manually marked in 3D-space with the Spots function. For the total numbers of cells reported per brain, average number of cells was first determined per slice in each animal, then multiplied by the average number of sections found per animal, by the number of serial collections analyzed, and by 2 for the hemispheres. The coordinates of all spots from each section were exported and used in Matlab.

### Computational Procedures and statistical analysis

To generate realistic distance distributions, we bootstrapped data using the following procedure. We randomly selected 74 brain sections of eight 2.5 months old animals, with replacements to generate resampled data. The random selection of sections was done to replicate the natural variability in cell numbers and DG geometry present across samples and sections. In each section, we randomly selected a subset of RGL GFAP^+^ cells to represent a sham BrdU^+^ cell distribution. The number of sham BrdU^+^ cells in the randomly selected subset was the same as the number of BrdU^+^ cells actually observed in the section. Because our resampling procedure was done with substantial subsampling, we chose cells without replacements, i.e. in each sham BrdU^+^ population in a slice, the same cell could not be selected more than one time. For both the BrdU^+^ and sham BrdU^+^ populations, we computed the nearest neighbor distance histograms (NNDH) (Fig. 1) and cell distance histograms (CDH). To replicate correlations present in the geometry of naturally occurring quiescent cell populations, we used the observed 3D coordinates of selected sham BrdU^+^ and 3D coordinates of observed BrdU^+^ cells in this calculation. This procedure was repeated for every section with BrdU^+^ cells and for each of the 74 randomly selected sections in the case of sham BrdU^+^ cells. The nearest neighbor distributions combined from all sections were then compared using the two-sided Kolmogorov-Smirnov test from the MATLAB package (Mathworks, Inc.).

To further compare distributions of both nearest and non-nearest neighbor distances (Fig. 1E,F), we repeated this resampling procedure with a ${N}_{tr}=1000$ times. For each of the iterations of bootstrap, we evaluated both NNDH and CDH. We then compared these histograms to the ones computed for the actual (non sham) BrdU+ cells. For each of the 5 μm bins, we computed two numbers ${f}_{ > }={N}_{ > }/{N}_{th}$ and ${f}_{ < }={N}_{ < }/{N}_{th}$, where ${N}_{ > }$ and ${N}_{ < }$ are the number of iterations of bootstrap out of ${N}_{tr}=1000$ in which the sham NNDH is above or below the actual one. We then computed the p-values for each bin $p=\,\min ({f}_{ > },{f}_{ < })$. We also repeated the procedure with CDH (Fig. 1F) for each of the 5 µm distance bins. We then tested the hypothesis that the distributions are different in each bin by using the FDR correction (mafdr function of MATLAB). For every bin and condition, we find all FDR q-values to be above 0.13 as indicated in Fig. 1E and F. This observation further confirms the results of the Kolmogorov-Smirnov test (Fig. 1).

To compare the NNDHs in young and adult animals, we generated sham 7 month-old animal cell distributions by subsampling the young animal data. First, we corrected the young data (P14, n = 4 mice, 27 sections) for an age-related 15% expansion. Second, for each 50 µm slice of the actual 7 month old animal data (31 sections, n = 4 mice), we selected a random slice of P14 data. The selection was made with replacements. We then randomly selected, without replacements, a set of cells in the young data slice that matched the number of cells in the adult data (31 sections from 27 collected from *n* = 4 P14 animals). Once the resampling was done for all slices, we computed the NNDH as described above and performed the Kolmogorov-Smirnov test. We then tested the detailed differences of distributions in different bins. To this end, we repeated the resampling procedure 1000 times and computed p-values for each 5 µm bin as described above. FDR procedure yielded q-values that were used to determine the statistical significance of the difference between the real and resampled data in individual bins, as described above.

To examine the regional differences in stem cell elimination, all sections from P14 and 7 months old mice were assigned to dorsal, intermediate and ventral DG according to the previously reported method used for coronal sections^27^. Coronal sections were referenced with sagittal sections using Brain Explorer® 3-D viewer integrated with Mouse brain atlas (2004 Allen Institute for Brain Science. Allen Mouse Brain Atlas. Available from: http://mouse.brain-map.org). Additionally, the positions of cells in the infrapyramidal blade (IB) and the suprapyramidal blade (SB) were defined. Overall, 11 sections with dorsal IB, 11 sections with dorsal SB, 15 sections with intermediate/ventral IB, and 15 section with intermediate/ventral SB were collected from P14 animals (*n* = 4) to obtain the actual distributions of distances between RGLs in the regions mentioned (Fig. 4, red). 16 sections with IB, 16 sections with SB, and 12 sections with intermediate/ventral IB, 19 sections with intermediate/ventral SB were collected from 7 month old mice (*n* = 4). These were used to randomly choose the number of sections matching those from P14 animals (the selection was made with replacements) and obtain 1,000 distributions for the resampled RGLs (Fig. 4, blue). In each iteration of such a resampling, the Kolmogorov-Smirnov p-values are randomly sampled variables. To evaluate the average Kolmogorov-Smirnov p-values for cell elimination in hippocampal regions, we averaged the logarithms of the p-values across 1000 comparisons between bootstrap resamples and actual data.

### Data availability

The data that support the findings of this study (i.e. images and coordinates of dividing RGLs and all RGLs) are available from the corresponding authors upon request. The codes used for bootstrap and calculation of the differences are available upon request from Dr. A. Koulakov (koulakov@cshl.edu).

## Electronic supplementary material

Supplementary Information
