# Supplementary material for: Spatial geometry of stem cell proliferation in the adult hippocampus
Source: Sci Rep. 2018 Feb 21;8:3444. doi: 10.1038/s41598-018-21078-6 (PMC5821870; doi:10.1038/s41598-018-21078-6)
Supplement: Supplementary file 1 — Supplementary Information [file 41598_2018_21078_MOESM1_ESM.pdf]

SUPPLEMENTARY INFORMATION

SPATIAL GEOMETRY OF STEM CELL PROLIFERATION IN THE ADULT HIPPOCAMPUS

Olga Mineyeva, Grigori Enikolopov, and Alexei A. Koulakov

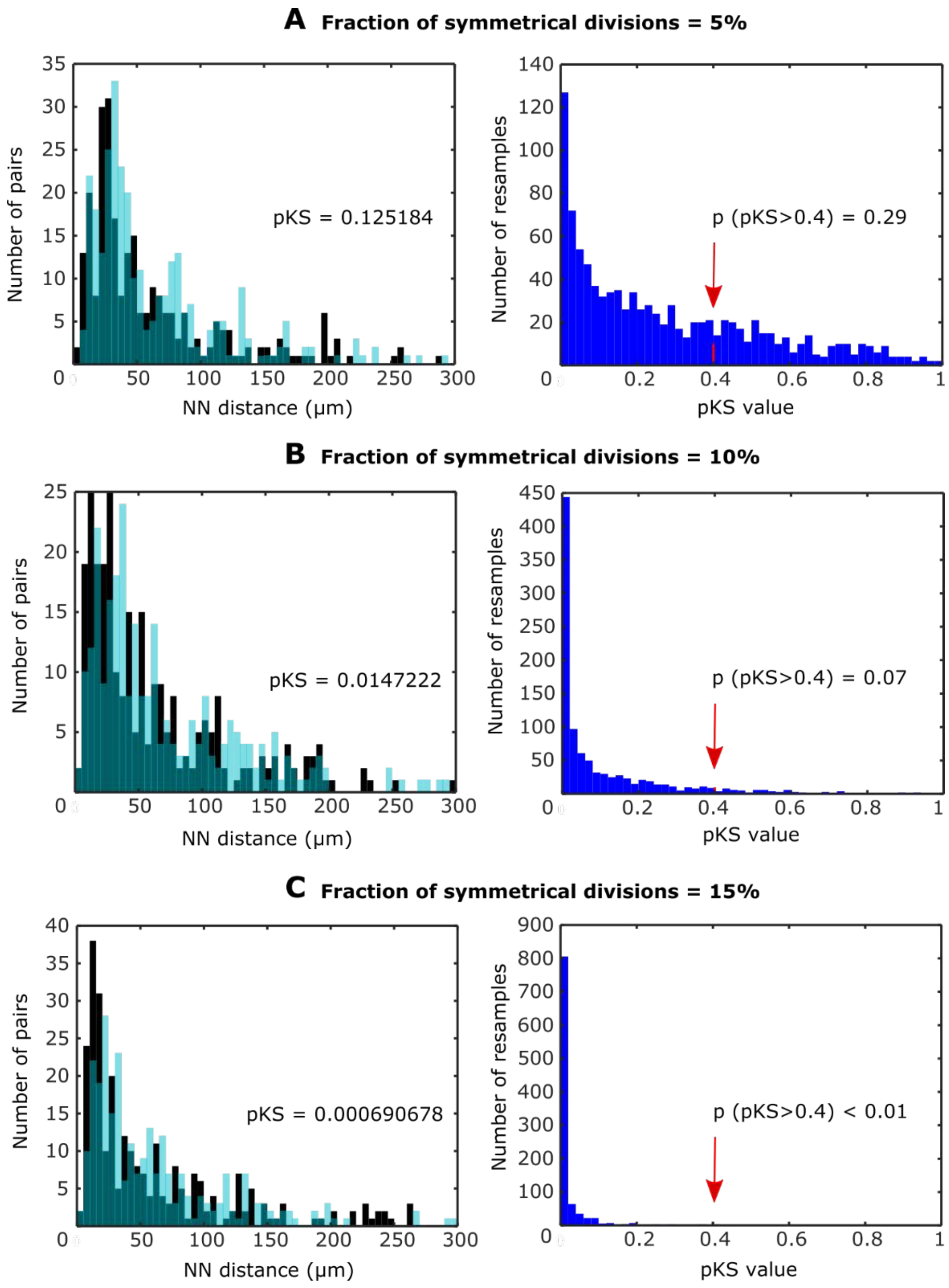

Figure S1. The sensitivity of RAM. We tested our technique by repeating our analysis in Fig. 1 with the certain number of symmetric divisions artificially introduced into the data. (**a**) When the fraction of symmetric divisions artificially introduced is 5%, observing similar actual and resampled nearest neighbor distributions, as in Fig.1 is very likely. $p>0.4$ is observed in 29% of cases. (**b**) For 10% of artificially introduced symmetric divisions, failure to detect them ($p>0.4$) is less likely (7%). (**c**) For 15% of symmetric divisions, observing $p>0.4$, as in our data is very unlikely (<1%). We conclude that 15% of symmetrical divisions is unlikely to be consistent with our data, while the borderline of 10% is consistent.

A

Age	Cell type	Average no. of sampled sections per DG	Total cells in sampled sections Mean, SD	Total cells per DG Mean, SD
2 weeks	RGLs	7	2301, 536	26356, 5018
2.5 months	RGLs	16	815, 62 *	8197, 195
	$BrdU^+$ RGLs	16	68, 15 *	777, 146
	Non-radial	8	15, 6	174, 77
	$BrdU^+$ Non-radial	8	6, 5	66, 54
7 months	RGLs	8.5	361, 48	3836, 354

B

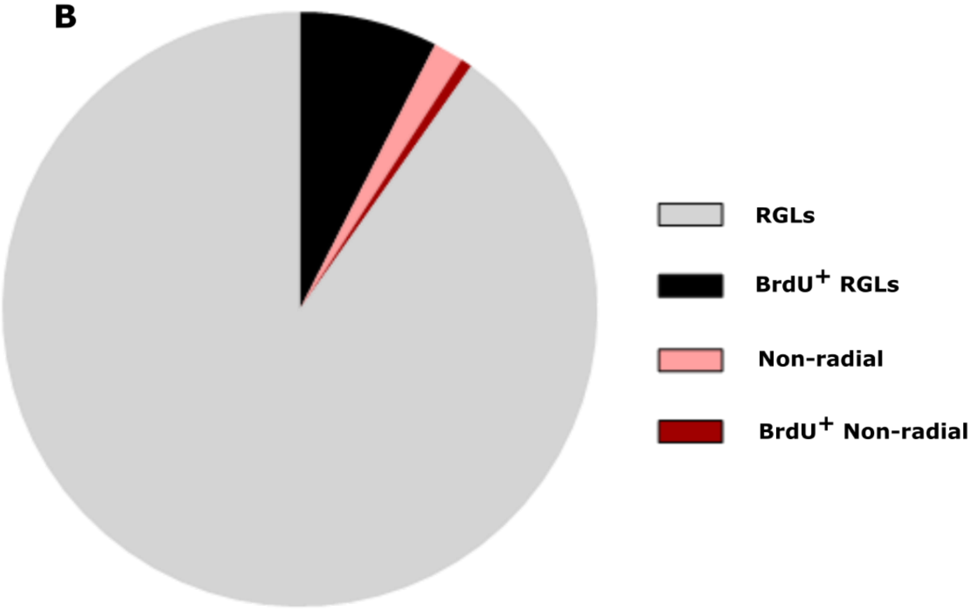

Figure S2. RGLs and non-radial GFP/GFAP-positive cells in the DG. (**a**) 4 animals were analyzed for each age. Estimation per DG presented as average number of cells per section in each animal multiplied by the average number of sections collected, by 6 (every sixth section was analyzed), and by 2 (for 2 hemispheres). * - One collection of 8 sections was used for each animal on average, the same sections were analyzed for non-radial cells. (**b**) Graphical representation of the data from the forth column, i.e. RGLs and non-radial GFP/GFAP-positive cells in 2.5-month-old animals.
